# Withholding planned speech is reflected in synchronized beta-band oscillations

**DOI:** 10.3389/fnhum.2015.00549

**Published:** 2015-10-12

**Authors:** Vitória Piai, Ardi Roelofs, Joost Rommers, Kristoffer Dahlslätt, Eric Maris

**Affiliations:** ^1^Donders Institute for Brain, Cognition and Behaviour, Radboud UniversityNijmegen, Netherlands; ^2^Knight Lab, Department of Psychology and Helen Wills Neuroscience Institute, University of California, BerkeleyBerkeley, CA, USA; ^3^Department of Psychology and The Beckman Institute for Advanced Science and Technology, University of IllinoisUrbana, IL, USA; ^4^Independent ResearcherBerkeley, CA, USA

**Keywords:** beta oscillations, conversation, delayed naming, dorsolateral prefrontal cortex, go/no-go, magnetoencephalography, synchronization, turn-taking

## Abstract

When engaged in a conversation, speakers sometimes have to withhold a planned response, for example, before it is their turn to speak. In the present study, using magnetoencephalography (MEG) outside of a conversational setting, we investigate the oscillatory brain mechanisms involved in the process of withholding a planned verbal response until it is time to speak. Our participants viewed a sequence of four random consonant strings and one pseudoword, which they had to pronounce when the fifth string (the imperative stimulus) was presented. The pseudoword appeared either as the fourth or fifth stimulus in the sequence, creating two conditions. In the withhold condition, the pseudoword was the fourth string and the verbal response was withheld until the imperative stimulus was presented. In the control condition, the fifth string was the pseudoword, so no response was withheld. We compared oscillatory responses to the withhold relative to the control condition in the time period preceding speech. Alpha-beta power (8–30 Hz) decreased over occipital sensors in the withhold condition relative to the control condition. Source-level analysis indicated a posterior source (i.e., occipital cortex) associated with the alpha-beta power decreases. This occipital alpha-beta desynchronization likely reflects attentional allocation to the upcoming imperative stimulus. Moreover, beta (12–20 Hz) power increased over frontal sensors. Source-level analysis indicated a frontal source (i.e., middle and superior frontal gyri) associated with the beta-power increases. We interpret the frontal beta synchronization to reflect a mechanism aiding the maintenance of the current motor or cognitive state. Our results provide a window into a possible oscillatory mechanism implementing the ability of speakers to withhold a planned verbal response until they have to speak.

## Introduction

Conversation is marked by a well-coordinated taking of turns between the listener and the speaker. Although occasionally a speaker will fail to withhold her response until it is her turn to speak, the vast majority of transitions between a speaker’s and a listener’s turns is smooth with minimal gap in between (e.g., Sacks et al., 1974; Stivers et al., 2009). So the ability of a speaker to withhold a planned response seems to be an important component of successful communication. Anecdotally, we are all familiar with the situation of feeling ready to say something, but doing so would cause our utterance to overlap with the speech of our conversation partner. Thus, we withhold our utterance until it is our turn to speak.

The fast and accurate transitions between speaker and listener roles in human conversation are an intriguing achievement considering that conversational turns are not fixed in length nor restricted to a particular phrase or syntactic construction (e.g., Sacks et al., 1974). The issue becomes even more interesting if one considers that a speaker may need about 600 ms to plan and begin to articulate a simple word (e.g., Indefrey and Levelt, 2004). Turn-taking studies have been conducted investigating what enables this precise timing of turn-taking in conversation (e.g., de Ruiter et al., 2006; Magyari and de Ruiter, 2012; Torreira et al., 2015). For example, Magyari et al. (2014) stated, “Given the latency of the speech production process, if speakers are going to come in on time, they must begin the production process well before the end of the other’s turn” (p. 2536; see also Levinson and Torreira, 2015). This points to the potential need to withhold a planned response: If a speaker begins the production process long before it is her time to speak, she may plan an utterance that cannot yet be articulated, as producing it would create overlap with the utterance of her interlocutor. In the present study, we investigate the speaker in a non-conversational setting with the objective of identifying the neurophysiological correlate of her ability to withhold speech.

Researchers have only recently started examining oscillatory brain activity associated with planning and producing speech (e.g., Gehrig et al., 2012; Herman et al., 2013; Jenson et al., 2014). These studies have found that desynchronization in the alpha and beta bands (7–30 Hz), localized to left inferior frontal, motor, and premotor cortex (e.g., Herman et al., 2013), precedes speech onset. This desynchronization has been interpreted in relation to the well-known alpha-beta desynchronization characteristic of preparation and execution of (hand) movements (e.g., Pfurtscheller and Lopes da Silva, 1999; for review Cheyne, 2013). The finding that speech planning has a neurophysiological signature akin to that of general motor preparation (i.e., alpha-beta desynchronization) is exciting because it allows us to link the neural mechanisms supporting speech production to mechanisms supporting other brain functions, such as motor control (see Piai et al., 2015, for further discussion).

However, speakers must also be able to withhold a verbal response that has already been planned. Very little is known about the neurophysiology of this ability. In particular, no study has yet examined oscillatory activity related to withholding a planned verbal response. Note that robust beta power increases have been shown for withholding a planned manual response (i.e., “postural maintenance”, see e.g., Engel and Fries, 2010; Kilavik et al., 2013; for reviews) Previous production studies employing electroencephalography and requiring participants to withhold a planned response have only reported event-related potentials (e.g., Jescheniak et al., 2003). Moreover, those studies were mainly concerned with addressing a psycholinguistic question, rather than with neurophysiological mechanisms of speech.

In the present study, we focus on the neurophysiology of withholding planned speech, as measured through brain oscillations. Although our paradigm does not capture the real dynamics of naturalistic human conversation, it does allow us to investigate a core component of speaking in such a setting. Our participants engaged in planning a verbal response that sometimes had to be withheld. On half of the trials, they planned a response but withheld it until a cue was presented (withhold condition). On the other half of the trials, the response could only be planned after the cue was presented (control condition). Using magnetoencephalography (MEG), we compared pre-speech oscillatory brain responses in these two conditions.

## Materials and Methods

### Participants

Fifteen native speakers of Dutch (6 male, mean age = 23 years, *sd* = 3.2) voluntarily participated in the experiment for monetary compensation or for course credits after providing written informed consent. The datasets of four additional participants were not analyzed due to excessive blinking resulting in the loss of a large number of trials (less than 70% of the trials remaining). The present experiment was approved by the Ethics Committee for Behavioral Research of the Social Sciences Faculty at Radboud University Nijmegen in compliance with the Code of Ethics of the World Medical Association (Declaration of Helsinki).

### Materials

A set of 204 pronounceable pseudowords was generated using WordGen (Duyck et al., 2004). All pseudowords had between two and ten orthographic neighbors and were of four, five, or six letters length (68 pseudowords of each length). Furthermore, a set of 204 random consonant strings was generated of four, five, and six characters length (68 strings of each) to serve as control items for the pseudowords. Finally, another set of 375 random consonant strings was generated of four, five, and six characters length (125 filler strings of each) to be presented in the first, second, and third position of the sequence.

### Design

Each pseudoword was paired with a control consonant string of the same length. Half of the pairs appeared in the withhold condition and the other half in the control, counterbalanced across participants. The 204 pairs were pseudo-randomized using Mix (van Casteren and Davis, 2006) with at most five consecutive trials of the same condition. For each trial, three consonant strings were selected at random (without replacement) from the 375 filler strings. One unique randomized list per participant was used.

### Behavioral Procedure

Participants were tested individually in an electrically, acoustically, and magnetically shielded room. The experimenter provided non-magnetic clothes to the participants. Participants were instructed to keep fixation on the center of the screen, to minimize (head) movement during the experimental blocks, and to blink only during the blinking intervals (see below).

In every trial, participants were presented with five strings, and they had to respond to the fifth one (i.e., the imperative stimulus). One of the five strings was a pseudoword that had to be pronounced. This pseudoword was either on the fourth position, in which case the pronunciation had to be withheld and the fifth string served as a go cue (the withhold condition), or it was on the fifth position, in which case it had to be pronounced immediately (the control condition). In the following, we will denote the fifth string as the *imperative stimulus*, because it triggers the pronunciation of the pseudoword. Thus, in the withhold condition, the imperative stimulus is a consonant string that serves as a go cue for the pronunciation of the pseudoword that is presented as the fourth string. In the control condition, the imperative stimulus is a pseudoword that serves both as a go cue and provides the content of the pronunciation. Speed as well as accuracy were emphasized. Participants then practiced the task with 15 trials. After that, they were brought to the shielded room.

**Figure 1:**
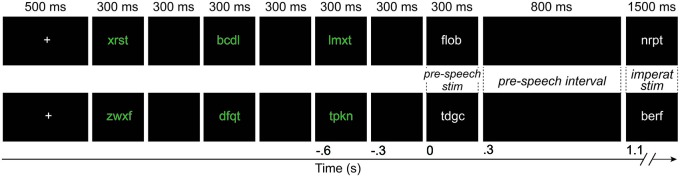
**An example of a withhold condition (upper) and a control condition (lower) trial**. The duration of each event is shown in milliseconds on top of the figure. The time line, relative to the presentation of the pre-speech stimulus, is shown on the bottom in seconds. The width of the black boxes is proportional to the duration of the events in the trial, except for the presentation of the imperative stimulus. The *** presented for 2 s at the end of the trial are not shown. Imperat, imperative; stim, stimulus.

Stimuli were presented through an overhead projector on a screen placed 90 cm in front of the participants. The stimuli were in Arial font, size 20. A trial began with a fixation cross presented for 500 ms. Three consonant strings were then presented in green ink for 300 ms, interleaved with a black screen for 300 ms. The fourth and fifth stimuli were presented in white ink. The fourth stimulus was presented for 300 ms, followed by a black screen of 800 ms. The fifth stimulus (imperative stimulus) was then presented for 1.5 s, followed by *** for 2 s, which was the blinking interval. The use of two colors was intended to better guide participants in differentiating between the three initial consonant strings (presented in green), and the pre-speech and imperative stimuli (in white), discouraging them to count the stimuli. Figure 1 presents an illustration of the trial structure. The 204 experimental trials were divided into four blocks with self-paced breaks in between.

### MEG Procedure

The MEG system (CTF VSM MedTech) contained 274 axial gradiometers. Pairs of Ag/AgCl-electrodes were used to record the surface electromyogram from the orbicularis oris muscle and the horizontal and vertical electro-oculogram (impedance <15 kΩ for all electrodes). Three localization coils were fixed to the nasion, right, and left ear canal to monitor the position of participants’ heads relative to the gradiometers. Head localization was performed in real time, with the head position re-adjusted when it deviated more than 9 mm from the initial position (Stolk et al., 2013). The data were low-pass filtered by an anti-aliasing filter (300 Hz cutoff), digitized at 1200 Hz, and stored for offline analysis. A microphone in the magnetically shielded room was connected to a computer, which recorded the vocal responses and controlled stimulus presentation with the software package Presentation (Neurobehavioral Systems). Anatomical T1-weighted magnetic resonance images (MRI) of the participants’ brains were acquired with a 1.5 T Siemens Magnetom Sonata system using a magnetization-prepared, rapid-acquisition gradient echo sequence.

### Response-time Analysis

Verbal responses were evaluated in real time. Responses containing disfluencies were marked, as well as responses initiated before the cue stimulus was presented (3.2% of the trials). Their corresponding trials were subsequently excluded from all analyses. Response times (RTs) were calculated manually using the speech waveform editor Praat (Boersma and Weenink, 2013) before the trials were separated by condition. The statistical analysis was conducted using R (R Core Team, 2014). Participants’ RTs were skewed. Given that the median is the best representative of central tendency with skewed data, participants’ median RTs were computed for each condition. Paired-samples *t*-tests on participants’ median RTs were used to evaluate the behavioral effect. Group RT distributions were also examined by rank-ordering the RTs for each participant, dividing them into 20% quantiles, and then computing quantile means.

### MEG and EMG Data Analysis

The analyses were performed using FieldTrip version 20130515 (Oostenveld et al., 2011) in MatlabR2014b. The MEG data were down-sampled offline to 600 Hz and segmented into epochs time-locked to the pre-speech stimulus, from 0.3 s before the pre-speech stimulus (corresponding to the beginning of the black screen) to 1.2 s post-stimulus (corresponding to 100 ms after the presentation of the imperative stimulus, see Figure 1). Since speaking causes artifacts that could potentially affect the MEG signal, all trials in which participants responded within 150 ms after cue onset were discarded from all MEG and EMG analyses.

#### MEG Preprocessing

All MEG epochs were inspected individually for artifacts. Excessively noisy channels were also removed. Artifact- and error-free data comprised on average 94 trials per condition.

##### Sensor-level analysis

Synthetic planar gradients were calculated (Bastiaansen and Knösche, 2000). Temporal smearing is an inherent property of time-resolved power estimation. Accordingly, signal components elicited by the imperative stimulus (and therefore also by the participants’ initiation of speech) will affect power estimates for time intervals in the pre-speech interval. However, we do not suffer from smearing if we calculate the time-averaged power over the pre-speech interval (0.3–1.1 s after the pre-speech stimulus), and here we did this using multitaper-based spectral estimation. This method of spectral estimation allows for a precise control of the spectral smoothing. We estimated power between 5–30 Hz with 2 Hz spectral smoothing (i.e., 1 Hz above and 1 Hz below) over the pre-speech interval (0.3–1.1 s after the pre-speech stimulus). The data in the pre-speech interval was multiplied with discrete prolate spheroidal sequences as tapers and the Fourier transform was taken from the tapered signal. The power estimates were then averaged over trials for each condition and each participant.

Differences between the conditions were evaluated statistically using a non-parametric cluster-based permutation test (Maris and Oostenveld, 2007) applied to power as a function of frequency (8–30 Hz) and space (the MEG sensors). Given that the low-frequency range (5–7 Hz) was heavily contaminated by myogenic artifacts (see “Frontal Beta Power Increases are not due to Myogenic Artifacts” Section below), we restricted the statistical analysis to 8–30 Hz. For the statistical test, all parameters were the default settings of the Fieldtrip toolbox (version 20130515), except for the following parameters. Spatial clustering was performed on the basis of a neighborhood structure in which sensors had on average six neighbors. Only the sensors that were available for all participants were entered in the analyses (260 in total).

##### Source-level analysis

First, for each participant, the anatomical MRI was segmented using SPM8^1^, which was then used for constructing a corrected-sphere model of the inside of the skull (the volume conduction model, Nolte, 2003). Next, the participant-specific MRI was first warped to a template MRI (Montreal Neurological Institute (MNI), Montreal, QC, Canada) and then the inverse of that warp was applied to the dipole grid (a 3D grid with 1 cm resolution). This step yielded a grid in MNI coordinates for every participant, allowing us to directly compare grid points across participants in MNI space. The volume conduction model was then used to compute the lead field matrix for each grid point in the source model (Nolte, 2003).

Source-level power was estimated in the pre-speech interval (i.e., 0.3–1.1 s) using the dynamic imaging of coherent sources method (Gross et al., 2001). The sensor-level cross-spectral density matrix was computed from the data of the two conditions combined centered at 16 Hz (with 4 Hz spectral smoothing above and below) using discrete prolate spheroidal sequences as tapers. This frequency range was selected on the basis of the sensor-level results (see “Cortical Signatures of Planning and Withholding Planned Speech: Frontal Beta-power Increases and Posterior Alpha-Beta Power Decreases” Section below). The cross-spectral density matrix was then used together with the leadfields to compute the common spatial filters at each location of the 3-dimensional grid. The common spatial filters were applied to the Fourier transformed data from each condition separately to yield source-level spectral power estimates for each grid point in each condition. These power estimates were then averaged over the trials of each condition for each participant. Relative power change was calculated as the difference between the power in the two conditions divided by their average power. The differences in spectral power between conditions were evaluated using a non-parametric cluster-based permutation test (Maris and Oostenveld, 2007), resulting in a cluster of adjacent cortical locations exhibiting a similar difference across conditions (Fieldtrip toolbox, version 20130515, all parameters set to default).

##### EMG preprocessing and analysis

We analyzed the EMG to ensure that participants started planning their responses before the imperative stimulus. In that case, mouth muscles such as the orbicularis oris should show increased activity prior to the onset of the imperative stimulus. For one participant, EMG recordings failed so this analysis comprised 14 participants. For the EMG analysis, the EMG data were high-pass filtered offline at 15 Hz (Butterworth two-pass filter of 6th order, FieldTrip default settings) prior to segmentation (see van Boxtel, 2001, for a motivation of the cutoff frequency). The EMG was then Hilbert-transformed and rectified. The resulting signal was then segmented into epochs time-locked to the pre-speech stimulus, from 0.6 s before to 1.5 s after the pre-speech stimulus. Finally, the EMG was averaged over trials per participant for each condition separately. To test statistically whether EMG amplitude differed between conditions before the pre-speech stimulus, a non-parametric cluster-based permutation test was used (with 1,000 random permutations). On the basis of temporal adjacency, clusters exhibiting a similar difference between conditions were identified by means of dependent-samples *t*-tests thresholded at an alpha level of 0.05.

## Results

### Planned Responses are Articulated Earlier

**Figure 2:**
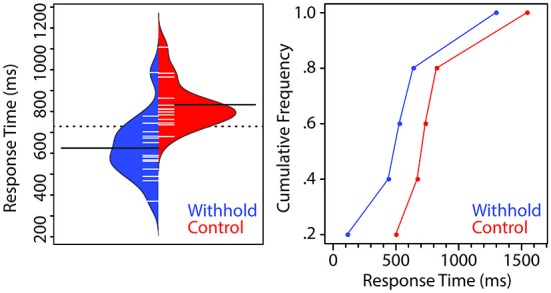
**Left**. Beanplot of the group-average response times (RTs) for the withhold (blue) and control (red) conditions. The dashed line indicates the group mean whereas the two filled lines indicate the mean of each condition. Each short line represents the median RT of each participant. **Right**. Cumulative distribution of participants’ RTs as a function of condition.

Figure 2 (left panel) shows a bean plot of the participants’ RTs (Kampstra, 2008), with the dashed line indicating the group mean and the two filled black lines indicating the mean of each condition. Each short white line represents the median RT of one participant for that condition. The cumulative distribution of participants’ RTs as a function of condition is shown in the right panel. Verbal responses were on average 208 ms faster in the withhold than in the control condition, *t*_(14)_ = 7.86, *p* < 0.001, 95% CI (151, 264). The cumulative RT distribution shows that the effect is the result of a shift of the entire distribution as a function of whether participants could prepare their responses before the cue or not. Moreover, the larger difference between the two conditions for the 20% fastest responses is possibly due to the fact that the inter-stimulus interval preceding the pre-speech stimulus is fixed. As such, participants are more likely to predict exactly when to speak.

### Planning and Withholding Speech Increases EMG Activity

**Figure 3:**
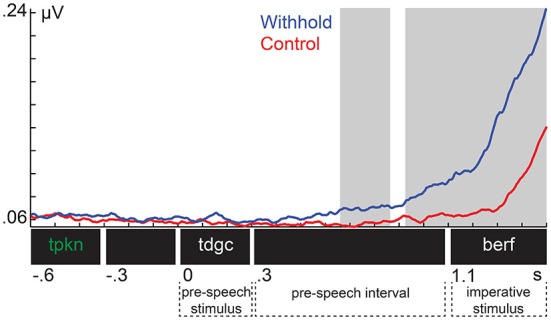
**Group-level electromyogenic activity from the orbicularis oris muscle for the withhold (blue) and control (red) condition**. Shaded areas indicate the time intervals associated with the significant clusters.

Figure 3 shows the EMG from the orbicularis oris muscle for each condition. Shaded areas indicate the time intervals associated with the significant clusters. The EMG was increased in the withhold condition relative to control already during the pre-speech interval, and this difference increased further after the imperative stimulus was presented. These observations were confirmed by the cluster-based permutation test, which revealed two temporal clusters that exhibited a larger amplitude in the withhold than in the control condition (*p* = 0.009 and *p* < 0.001, respectively). These clusters were detected between 660 ms and 862 ms (left shaded area), and from 925 ms until the end of the segment (i.e., 1500 ms, right shaded area). Thus, we have a physiological indication that participants planned their responses in the withhold condition already prior to the imperative stimulus.

### Cortical Signatures of Planning and Withholding Planned Speech: Frontal Beta-Power Increases and Posterior Alpha-Beta Power Decreases

**Figure 4:**
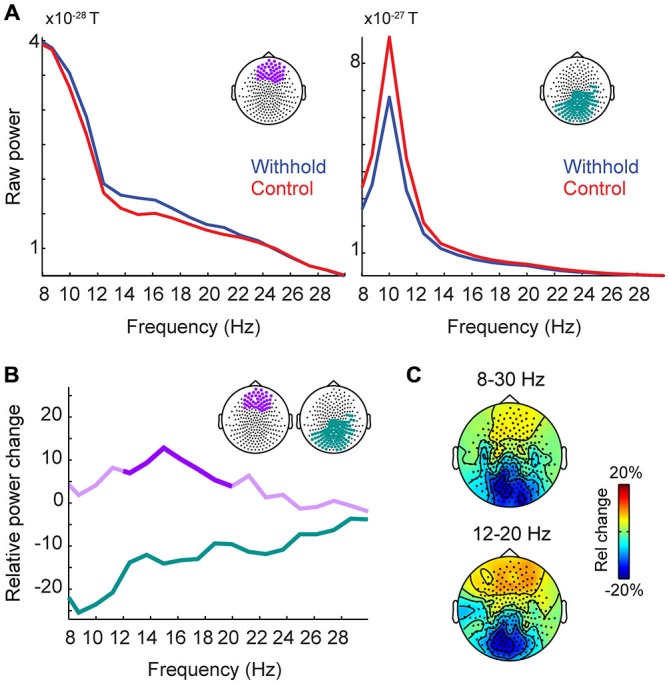
**Group-level raw power and relative power changes (withhold vs. control) during the pre-speech interval. (A)** Group-level raw power spectra averaged over a group of frontal sensors (left panel) and posterior sensors (right panel) for the withhold (blue) and control (red) conditions. **(B)** Group-level relative power spectra averaged over a group of frontal (purple) and posterior (green) sensors as shown in the top right corner of panel **(B)**. Significant frequency ranges are indicated by stronger colors (frontal sensors: 12–20 Hz; posterior sensors: 8–30 Hz). **(C)** Group-level topographical maps of the relative power changes for two frequency ranges (8–30 Hz and 12–20 Hz), indicated on top of each map. Rel, relative.

We statistically compared the withhold and the control condition with respect to time-averaged power (shown in Figure 4A) as a function of both frequency and space (sensor location). In a cluster-based permutation test, two clusters with significant *p* values were observed, one over frontal and one over posterior sensors. Figure 4A shows the power spectra for each condition during the pre-speech interval averaged over the significant frontal and posterior sensors shown on top of each spectrum. Figure 4B shows the relative power changes between the withhold and control conditions during the prespeech interval for the significant sensors. Over frontal sensors, power increased in the withhold relative to the control condition (withhold > control, between 5–13%) in the 12–20 Hz range (*p* = 0.012). This range is indicated by the strong purple color in Figure 4B. Over posterior sensors, power decreased in the withhold relative to the control condition (withhold < control, between 5–25%) in the 8–30 Hz range (*p* < 0.001). Figure 4C shows the topographical maps of the relative power changes for two frequency ranges, indicated on top of each map.

**Figure 5:**
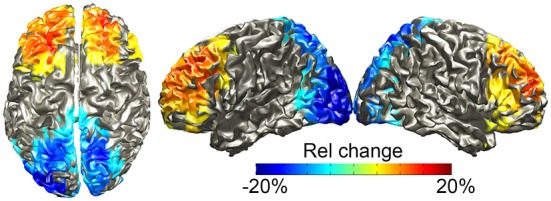
**Group-level source localization of the power differences (withhold vs. control) between 12–20 Hz during the pre-speech interval**. The color bar shows relative (rel) power changes, masked by the statistically significant clusters.

We source-localized both effects during the pre-speech interval using a frequency-domain beamformer analysis in the 12–20 Hz range, since this frequency range was optimal for the frontal power increases while capturing both the posterior power decreases and the frontal increases. We also statistically compared the withhold and the control condition at the source level using a cluster-level permutation test. Figure 5 shows the results, masked by the statistically significant clusters, with the color scale indicating the percentage change in power. The results parallel those of the sensor-level analysis: one positive cluster (withhold > control, *p* = 0.005) over bilateral frontal areas, localized to the superior and middle frontal gyri, and inferior frontal gyrus albeit less strong, and one negative cluster (withhold < control, *p* < 0.001) over bilateral occipital cortex. When source localizing the effects in the 8–30 Hz range, very similar results were obtained to what we present here.

**Figure 6:**
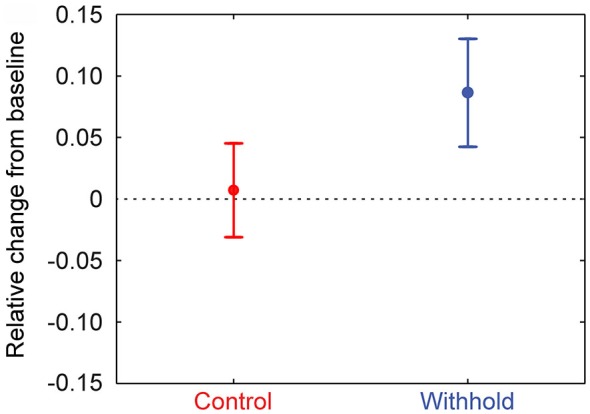
**Group-level mean frontal beta-power (12–20 Hz) during the pre-speech interval for the control (left) and withhold (right) condition relative to the baseline period**. Error bars indicate 95% confidence intervals.

To assess whether the frontal beta power differences during the pre-speech interval are due to a pattern of synchronization or desynchronization relative to baseline, frontal beta power (i.e., 12–20 Hz, statistically significant frontal sensors) was normalized to a baseline period (−0.3–0 s) for each participant. Participants’ normalized mean frontal beta-power and 95% confidence intervals are shown in Figure 6 for each condition separately. It is clear from the figure that frontal beta-power increases relative to the baseline period in the withhold condition, but remains similar to baseline levels in the control condition (the dashed lines indicate no (0) change from baseline). Participants’ normalized frontal beta-power was assessed statistically by means of a *t*-test against zero in each condition at an alpha-level of 0.025 to correct for two comparisons. In the withhold condition, frontal beta-power increased during the pre-speech interval relative to baseline, *t*_(14)_ = 4.22, *p* < 0.001. In the control condition, frontal beta-power was not significantly different from baseline, *t*_(14)_ = 0.40, *p* > 0.696. Finally, a paired-sample *t-test* indicated that the baseline normalized frontal beta-power was larger in the withhold (mean: 0.086) than in the control (mean: 0.007) condition, *t*_(14)_ = 3.50, *p* = 0.004.

#### Frontal Beta Power Increases are not Due to Myogenic Artifacts

Given that the EMG was increased for the withhold relative to the control condition already during the pre-speech interval, it is important to assess whether the power increases below 8 Hz and between 12–20 Hz are caused by myogenic artifacts. Below, we evaluate this possibility for each of these frequency bands.

**Figure 7:**
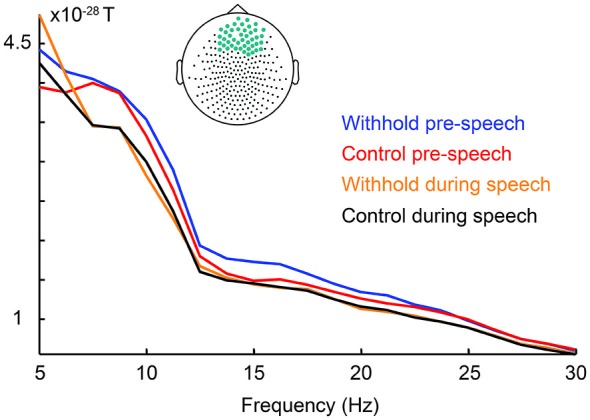
**Group-level raw power spectra as a function of condition during the pre-speech interval (withhold: blue; control: red) and during speech (withhold: orange; control: black) averaged over the frontal sensors indicated on top**.

Firstly, if myogenic activity would explain the beta effect prior to speech, then beta power should be stronger during speech, when myogenic activity is greatest, than preceding speech. Figure 7 shows the power spectrum for each condition pre-speech and during speech averaged over the frontal sensors indicated in light blue (i.e., the statistically significant frontal sensors). Contrary to the prediction, frontal beta power was lower during speech (orange and black lines) than in the pre-speech interval (blue and red lines) between 12–20 Hz. This observation was confirmed by a repeated measures analysis of variance on the frontal beta-band power as a function of condition (withhold vs. control) and interval (pre-speech vs. during speech) at an alpha level of 0.0125 to correct for four comparisons. Frontal beta power was lower during speech than in the pre-speech interval, as indicated by a main effect of interval, *F*_(1,15)_ = 20.55, *p* < 0.001. Condition and interval interacted, *F*_(1,15)_ = 18.60, *p* < 0.001, indicating that power was lower during speech than preceding speech for the withhold condition, *F*_(1,15)_ = 36.76, *p* < 0.001, but statistically similar for the control condition, *F*_(1,15)_ = 5.49, *p* = 0.035. With respect to the 5–7 Hz range, Figure 7 suggests that frontal power is similar pre-speech and during speech for both conditions. If 5–7 Hz frontal power during speech, when myogenic activity is greatest, is as high as preceding speech, it would indicate that the 5–7 Hz range is likely contaminated with myogenic activity. A repeated measures analysis of variance was conducted on the frontal power in the 5–7 Hz range as a function of condition (withhold vs. control) and interval (pre-speech vs. during speech) at an alpha level of 0.0125 to correct for four comparisons. Frontal power in the 5–7 Hz range was not statistically different during speech from preceding speech, *F*_(1,15)_ < 1, nor different between conditions, *F*_(1,15)_ = 4. 20, *p* = 0.063. Condition and interval did not interact, *F*_(1,15)_ < 1. Thus, we can conclude that the frontal power increases between 12–20 Hz during the pre-speech interval are not caused by myogenic artifacts. The frontal power increases between 5–7 Hz, however, are likely caused by muscle activity.

## Discussion

In the present study, we investigated the neurophysiology of withholding a planned verbal response through brain oscillations. It has been argued that neuronal oscillations may provide the key to understanding neuronal computations. Under this view, relating neurophysiological signatures in linguistic tasks to the signatures of other cognitive processes could help us understand language function in the context of more basic neurophysiological principles implemented in the brain (see for examples, Piai et al., 2014; Friederici and Singer, 2015).

Preceding the imperative stimulus (during the pre-speech interval), alpha-beta power (8–30 Hz) decreased 5–25% in the withhold relative to the control condition. This power decrease was restricted to occipital sensors and localized mainly to the occipital cortex bilaterally. By contrast, relative beta (12–20 Hz) power increased 5–13% over frontal sensors and was localized to a frontal source (middle and superior frontal gyri, and partly inferior frontal gyrus). Moreover, the EMG recorded from the orbicularis oris muscle was already increased for the withhold relative to the control condition during the pre-speech interval, confirming that participants prepared their responses. Below, we discuss the oscillatory effects in more detail.

The most robust oscillatory signature of preparing to speak is alpha-beta desynchronization in speech motor areas such as left inferior frontal cortex and ventral motor and premotor cortex (e.g., Salmelin and Sams, 2002; Saarinen et al., 2006; Herman et al., 2013; Jenson et al., 2014; Piai et al., 2015). Our results of beta synchronization in superior and middle frontal gyri when withholding a planned verbal response are clearly different from the signature of speech preparation.

An interesting parallel with our beta synchronization effect can be found in instructed delay tasks, such as go/no-go. In a review of these tasks, it was noted that beta synchronization is commonly observed during an interval of stimulus processing while overt movement is withheld until the go signal (Kilavik et al., 2013). This interval in go/no-go tasks is equivalent to our pre-speech interval. In the literature beta synchronization has been found not only over sensorimotor cortex but also extending further into the entire frontal lobe (see Kilavik et al., 2013 for a review of these findings). This suggests that a similar spatial-spectral pattern underlies withholding speech and withholding other types of overt movement. The functional role of this beta synchronization while overt movement is withheld, however, has not been well specified (see for discussion Kilavik et al., 2013). A tentative functional explanation for this beta synchronization may be found in the proposal of Engel and Fries (2010). On their account, if the current sensorimotor or cognitive state has to be maintained, beta activity is increased. In fact, these authors explicitly predict that activity in the beta band should be increased “during delay-periods where the cognitive set has to be maintained following a cue” (p. 160). This prediction fits with our observation of beta-power increases in the withhold condition during the interval when participants prepare but do not execute their verbal responses. Presumably, in this period, the current motor or cognitive state has to be maintained to enable successful speech production.

The maintenance of the sensorimotor state modulates activity in sensorimotor brain regions (see for review, Engel and Fries, 2010). In our case, the sensorimotor areas associated with speech planning would be left inferior frontal cortex and ventral motor and premotor cortex (e.g., Salmelin and Sams, 2002; Saarinen et al., 2006; Herman et al., 2013; Jenson et al., 2014). Yet, the beta-power increases we observed were more prominent in bilateral superior and middle frontal gyri, less so in bilateral inferior frontal gyrus, and (statistically and descriptively) absent in ventral motor and premotor cortex. It could be the case that cognitive-set maintenance would be subserved by dorsolateral prefrontal cortex (e.g., Petrides, 2005; Buschman et al., 2012; Stoll et al., 2015), compatible with our source localization to superior and middle frontal gyri. Modulations of frontal beta-band oscillations have also been found in a study manipulating the demands for cognitive control (Stoll et al., 2015), which is possibly involved in our task. Future studies will hopefully clarify these issues.

Modulations of alpha and beta oscillatory power often reflect expectation and prediction (e.g., van Ede et al., 2010; Arnal and Giraud, 2012; Pomper et al., 2015). Most relevant to the present study, alpha desynchronization in occipital cortex in expectation of a visual stimulus is a well-known finding (e.g., Foxe et al., 1998; Worden et al., 2000; Sauseng et al., 2005; Romei et al., 2010). Activity during our pre-speech interval is likely to encompass the anticipation of (or attention towards) the visually presented imperative stimulus, as well as preparation of the spoken response. The occipital source of our alpha-beta desynchronization speaks in favor of a visual attention interpretation, rather than preparation of the spoken response. Decreases in pre-stimulus posterior alpha-power have been often associated with improvements in visual perception (e.g., van Dijk et al., 2008; Jensen and Mazaheri, 2010; Jensen et al., 2012). There are at least two possibilities regarding attentional differences between the two conditions. One possibility is that in the control condition participants have to maximize visual processing to perceive the imperative stimulus and process its content, which is necessary for articulating the target. Another possibility is that in the withhold condition, the imperative stimulus is treated as a go signal and participants need to be maximally sensitive to it in order to respond as fast as possible upon its presentation. Under the assumption that alpha-band desynchronization reflects improved visual processing, or enhanced excitability of visual cortex (Lange et al., 2013), the first possibility would predict power decreases in the control relative to the withhold condition. However, this is the opposite of what we found. Thus, the power decreases are consistent with the second possibility, that is, the participants trying to be maximally sensitive to the imperative stimulus to respond as fast as possible after having prepared their responses.

It can be argued that the difference between the withhold and control conditions in the pre-speech interval is due to a different working-memory demand. Whereas in the control condition, participants were simply waiting for the imperative stimulus, in the withhold condition, they were maintaining the pseudoword in working memory. Although this hypothesis is compatible with our results, it is unclear whether it can fully account for our frontal beta synchronization effect. Firstly, the predominant oscillatory responses associated with working-memory maintenance are not within the frequency range in which we found power increases (12–20 Hz). In the working memory literature (Roux and Uhlhaas, 2014), oscillations between 4–13 Hz (theta and alpha) and above 30 Hz (gamma) have been associated with working-memory maintenance. Notably, the almost complete absence of beta-band effects in the working-memory literature has led some to question whether beta-band activity is even relevant for working memory (Roux and Uhlhaas, 2014). Moreover, if found, beta-band activity during working-memory maintenance tends to localize to posterior, rather than frontal, brain areas (see for review, Roux and Uhlhaas, 2014). Some form of working memory retention is inevitably involved in the act of withholding speech. In the present study, we did not intend to distinguish between this specific form of working memory retention and other forms (such as those that do not involve motor programming).

Furthermore, it can be argued that the fixed timing of stimulus presentation is a confound in our study because participants learned the timing of stimulus presentation, increasing their expectations. Importantly, however, the fixed timing of presentation was the case for both conditions. Thus, although the expectation of when stimuli will be presented plays a role in our task, it cannot exaplain the observed spectral differences between the two conditions.

In summary, when participants planned a verbal response and withheld it until an imperative stimulus was presented, beta-power (12–20 Hz) increases were observed in frontal brain areas relative to a control condition not involving speech planning and retention. For the same comparison during the same interval, power decreased over a broad range of frequencies (8–30 Hz) in occipital cortex. Both posterior alpha- and beta-power decreases and frontal beta-power increases are comparable to findings in other cognitive tasks not employing language or verbal responses. In keeping with the extant literature, we interpret our beta-power increases in relation to the maintenance of a cognitive (and possibly motor) set during the pre-speech interval. Altogether, these results suggest that a speaker’s ability to plan and withhold speech relies on similar neurophysiological computations as other cognitive functions outside of the language domain.

## Author Contributions

Conceptualized and designed the experiment (VP, AR, JR, EM); acquired the data (VP, KD); analyzed the data (VP, KD); wrote the paper (VP, AR, JR, KD, EM). All authors have approved the final version of the paper and agree to be accountable for all aspects of this work.

## Conflict of Interest Statement

The authors declare that the research was conducted in the absence of any commercial or financial relationships that could be construed as a potential conflict of interest.

## Footnotes

^1^http://www.fil.ion.ucl.ac.uk/spm/
